# Systematic Literature Review and Meta-Analysis of the Relationship Between Polyunsaturated and Trans Fatty Acids During Pregnancy and Offspring Weight Development

**DOI:** 10.3389/fnut.2021.625596

**Published:** 2021-03-25

**Authors:** Xuan Ren, Birgitta Lind Vilhjálmsdóttir, Jeanett Friis Rohde, Karen Christina Walker, Suzanne Elizabeth Runstedt, Lotte Lauritzen, Berit Lilienthal Heitmann, Ina Olmer Specht

**Affiliations:** ^1^Research Unit for Dietary Studies at the Parker Institute, Bispebjerg and Frederiksberg Hospital, Copenhagen University Hospital, Copenhagen, Denmark; ^2^Department of Nutrition, Exercise and Sports, Paediatric and International Nutrition, University of Copenhagen, Copenhagen, Denmark; ^3^Sydney Medical School, The Boden Institute of Obesity, Nutrition, Exercise, and Eating Disorders, Sydney University, Sydney, NSW, Australia; ^4^Section for General Medicine, Department of Public Health, University of Copenhagen, Copenhagen, Denmark

**Keywords:** N-3 LCPUFA, TFA, pregnancy, infant, birth weight, weight in childhood, BMI in childhood

## Abstract

Eicosapentaenoic acid (EPA), docosahexaenoic acid (DHA), and trans fatty acids (TFAs) may have an impact on offspring weight development. We conducted a systematic review and meta-analysis according to PRISMA guidelines to evaluate whether levels of these fatty acids during pregnancy influenced offspring weight development. Randomized controlled trials (RCTs) with DHA and/or EPA supplementation or cohort studies, which examined levels of DHA, EPA, or TFAs in maternal or neonatal blood samples and recorded offspring weight, were included. Overall, 27 RCTs and 14 observational studies were identified. The results showed that DHA and/or EPA supplementation doses >650 mg/day resulted in slightly higher birth weight (MD 87.5 g, 95% CI 52.3–122.6, *n* = 3,831) and combined BMI and BMI *z* score at 5–10 years (SMD 0.11, 95% CI 0.04–0.18, *n* = 3,220). These results were rated as moderate quality. Results from the observational studies were generally inconsistent. High TFA levels during pregnancy seemed to be associated with lower birth weight. Finally, this review and meta-analysis supports a relationship between high maternal or neonatal DHA and/or EPA levels and higher offspring birth weight and weight in childhood. More high-quality long-term studies are still needed.

## Introduction

Infant birth weight and the risk of macrosomia (birth weight above 4,000 g) have increased over the last decades in several industrialized countries (1–3). High birth weight is related to a risk of adverse outcomes at delivery, perinatal mortality, and obesity later in life (4). On the other hand, low birth weight also increases the risk of infant mortality and has negative long-term health consequences, including subnormal growth, non-communicable diseases, poor neurodevelopment, and lower academic achievement (5–8).

Fetal growth and birth weight are determined by several factors (9), of which maternal dietary intake is considered to play a key role (10). Adequate dietary intake, particularly intake of fatty acids, is important during pregnancy. Fatty acids provide most of the energy that is supplied to the fetus via the placenta, which will obviously affect energy metabolism and storage (11). Furthermore, specific fatty acids, like the n-3 long-chain polyunsaturated fatty acids (LCPUFA), eicosapentaenoic acid (EPA), and docosahexaenoic acid (DHA), have been shown to have an impact on duration of gestation and birth weight (12). Previous studies from the Faroe Islands suggest that DHA and/or EPA can increase birth weight by prolonging gestation periods and increasing the fetal growth rate (13, 14). During pregnancy, the demand for n-3 LCPUFA is higher than during other periods (15), and guidelines specify that pregnant women should consume more than 300 mg of DHA+EPA per day, of which at least 200 mg should be DHA (16–18). However, most pregnant women do not meet the EPA+DHA intake recommendation (19).

Eight previous systematic reviews of randomized clinical trials (RCTs) with n-3 LCPUFA (most included studies investigated the effect of DHA and/or EPA) supplementation during pregnancy and childhood growth have however shown conflicting results (12, 20–26). Most of the reviews included RCTs that examined effects of n-3 LCPUFA supplementation already during pregnancy, while two of the reviews included RCTs in which the intervention started during pregnancy and lactation (21, 24). Meta-analyses in four of the reviews showed increased birth weight in the DHA- and/or EPA-supplemented groups, and the mean difference between intervention groups and control groups ranges from 42.2 to 122.1 g (12, 20, 21, 26). One review found that increase in birth weight in DHA and/or EPA in the supplemented group might be caused by an increase in the length of gestation (12). However, other meta-analyses found no association with birth weight (22, 23), BMI, or BMI *z* score measured between 1 and 19 years of age (12, 21, 24, 25). The meta-analyses in the eight systematic reviews were based on different numbers of RCTs—from 6 to 43. Only one of the previous systematic reviews investigated effects among women with healthy pregnancies only (22), whereas six included RCTs with focus on high-risk pregnancies (allergic disease, gestational diabetes, obesity, and preterm birth depressive symptoms) (12, 20, 21, 23, 25, 26) or RCTs with intervention starting during lactation (24). The DHA and/or EPA dose in the RCTs varied, and it is likely that this would be of importance, but the effect of dose was only investigated in one of the previous reviews (12). This review reported high doses of DHA and/or EPA (more than 500 mg/day) during pregnancy associated with higher birth weight than low doses (12). There are an additional number of observational studies in the field, but to date, no systematic reviews have synthesized the collective evidence for an association between maternal n-3 LCPUFA status during pregnancy and birth outcomes and child growth. Because of the mixed results shown in the meta-analysis and the unknown effect of various doses, it is necessary to continue to perform further studies as well as systematic reviews and meta-analysis of results from both RCTs and observational studies.

There are two major sources of trans fatty acids (TFAs), industrial TFA (iTFA), and ruminant TFA (rTFA). iTFAs are formed by hydrogenation of vegetable oils, and rTFAs are produced by bio-hydrogenation in the rumen of animals (27). TFAs have been shown to be inversely associated with n-3 LCPUFA in maternal serum during pregnancy (28), and there is evidence of trans-placental transport of TFAs and presence of TFAs in umbilical cord blood (28, 29). It has also been hypothesized that TFAs could have a negative impact on fetal growth and development (30, 31); e.g., it has been suggested that higher levels of TFAs in maternal plasma are associated with low birth weight and short duration of gestation (32, 33). High intake of total TFAs during pregnancy may also influence growth patterns during infancy and childhood (34), but this has generally not been thoroughly examined. Partially hydrogenated fats, meat, and dairy fats are the main sources of TFAs (35), and according to the WHO recommendations, the intake of both iTFAs and rTFAs should be <1% of the total energy intake (36).

The aims of this systematic literature review and meta-analysis were two-fold, e.g., to synthesize study results from (1) RCTs on the effect of n-3 LCPUFA supplementation and the dose, in healthy pregnant women, on both birth weight and weight during childhood, and (2) observational studies examining associations between n-3 LCPUFA or TFAs from maternal or neonatal blood or placenta tissue and birth weight and weight during childhood.

## Materials and Methods

This systematic literature review and meta-analysis was performed according to Preferred Reporting Items for Systematic reviews and Meta-Analysis (PRISMA) and registered at PROSPERO (no. CRD42019115892). In the analyses, we stratified the included RCTs according to the doses provided to the intervention groups. We stratified doses into three levels: below 300 mg/day, 301–650 mg/day, and more than 650 mg/day. This stratification was based on the recommended intake of n-3 LCPUFA by WHO, of which pregnant women should consume more than 300 mg/day DHA+EPA (17). For the present review, we divided child weight according to the age groups 0–4 years and 5–10 years of age, using the BMI-for-age growth charts, which suggest that child BMI declines between 0 and 4 years of age but increases again after 5 years of age. The included observational studies were similarly focused on healthy pregnant women and on the associations between the outcomes specified for the RCTs and maternal or infant blood or placenta samples of n-3 LCPUFA and TFA intake. The results from the RCTs and observational studies were both included in the evaluation of the evidence for a potential causal relationship between intake of n-3 LCPUFA or TFAs during pregnancy and fetal and child growth.

### Search Strategy

Two databases, PubMed and EMBASE, were used to search for relevant articles in May 2019, using both MeSH terms and free terms. The following search terms were used: “Fatty Acids, Omega-3,” “Trans Fatty Acids,” “pregnancy,” “Infant, Low Birth Weight,” “Child Development,” “Birth Weight,” “Body Composition,” “Body Weight,” “Body Mass Index,” “Growth and Development,” “Growth,” “infant,” “child,” and “adolescent” (Supplementary Table 3). The titles and abstracts of studies were used to screen articles, which were not in line with the inclusion criteria. The actual selection was conducted based on a full text screening of the papers that passed the initial screening.

Both the literature search and the assessment of the search results were performed independently by two reviewers, XR and BV.

### Inclusion and Exclusion Criteria

We included RCTs and observational studies. The selection criteria were based on the predefined population, intervention, comparison, outcomes (PICO) model. All years of publication were eligible for inclusion. Languages were restricted to English and Chinese.

RCTs were included based on the following PICO criteria:

**Population**: Healthy pregnant women and children.

**Intervention**: DHA and/or EPA as supplement(s) during pregnancy.

**Comparison**: No treatment groups.

**Outcomes:** Primary outcome is offspring weight; secondary outcome is BMI (BMI, BMI *z* score, body composition, and fat distribution).

The included observational studies should:

1. Measure associations between levels of fatty acid and weight or BMI at different age groups (weight at birth, aged 0–4 years, and aged 5–10 years).
2. Associations between polyunsaturated and trans fatty acids consumption during pregnancy and offspring adiposity were presented based on fully adjusted models.
3. The sample tissue, from which levels of DHA and/or EPA were analyzed, should be maternal plasma or placental tissues.
4. The DHA content in the tissue should be expressed in percentage of total fatty acids.

We excluded cross-sectional studies with no control group, chart reviews, case series, commentaries (e.g., expert opinion, consensus statements), self-reported dietary information studies, and animal studies.

### Data Extraction

The two reviewers, XR and BV, independently used Covidence to extract data from eligible RCT studies. Disagreement was resolved through discussion. The included information from RCTs were authors, publication year, number of participants, weight, or BMI at different ages (weight at birth, age 0–4 years, and age 5–10 years) and supplementation doses. Afterwards, included RCTs were divided into different groups according to n-3 PUFA supplementation doses (0–300 mg/day, 301–650 mg/day, and more than 650 mg/day).

The included information from observational studies were authors, publication year, results from each observational study, number of participants, percentage of boy participants, beta coefficient (β), and 95% confidence intervals (95% CI) for associations between fatty acid levels and weight or BMI at different age groups (weight at birth, age 0–4 years, and age 5–10 years).

In case of identification of multiple reports of a single RCT or observational study, the publication with most completed data was included, and if all reports had complete information, then it was treated as a single study with references made to all the publications. When several publications from one trial reported at different ages or outcomes, these publications were included in different meta-analyses. Some trials reported birth weight and offspring weight or BMI in two different publications. These two publications were included in different meta-analyses. In addition, at age 0–4 years and age 5–10 years, most included studies investigated weight or BMI around 2 and 5 years of age, respectively.

### Statistical Analyses

Data from the RCTs or observational studies were quantitatively synthesized in a random effects meta-analysis to generate pooled effect estimates for birth weight or weight throughout childhood. The results from studies not possible to include in a meta-analysis were summarized narratively.

Continuous data from RCTs was analyzed by mean differences and 95% CI. According to the doses of fatty acid supplements from included RCTs, the meta-analysis was divided into three subgroups (0–300 mg/day, 301–650 mg/day, and more than 650 mg/day). The two kinds of supplementation (DHA or DHA+EPA) were investigated using subgroup analyses following doses. Both BMI and BMI z score reflect weight during childhood and standardized mean difference (SMD) is used when meta-analyses assess the same outcome but measure BMI in different ways (37, 38). Therefore, in the meta-analysis of BMI at age 5–10 years, we combined offspring BMI and BMI *z* score. For results from observational studies, adjusted β correlation and corresponding 95% CI between fatty acid levels and birth or child weight were used for all eligible studies. In addition, continuous outcomes were analyzed by β and 95% CI. Meta-analyses combined data and *I*^2^-values to quantify statistical heterogeneity, with an *I*^2^-value >50% considered to be substantial heterogeneity. We explored the possible cause(s) of heterogeneity when *I*^2^ was >50% by subgroup analyses.

Statistical analyses of RCTs were performed using Review manager 5.3 (RRID:SCR_003581), and statistical analyses of observational studies were performed using R 3.6.1.

### Study Quality Assessment

Two reviewers (XR and BLV) assessed the quality of each RCT using the Cochrane risk of bias tool (39). Discrepancies between the two reviewers were resolved through discussion.

XR and BLV also assessed the quality of each observational study criteria outlined by the Risk of Bias in Non-Randomized Studies of Intervention (ROBINS-I) tool (40). Discrepancies were resolved through discussion. A Directed Acyclic Graph (DAG) was used to identify potential confounders (Supplementary Figure 1). Predefined confounders were maternal BMI (41), maternal smoking (42), and socioeconomic status (43).

For both RCTs and observational studies, the Grading of Recommendation Assessment, Development and Evaluation (GRADE) methodology was used to assess the quality of the evidence, with the four possible ratings of results: very low, low, moderate, and high. Downgrading was done, using the standard definitions risk of bias, indirectness, inconsistency, imprecision, and publication bias. A funnel plot was used to assess publication bias when more than 10 studies were included. If <10 studies were included in the funnel plots, the power of the test for detecting asymmetry would be too low (39).

Inconsistency was identified by test heterogeneity. The *I*^2^-value was used to assess the degree of heterogeneity. If the *I*^2^-value was <50%, heterogeneity was considered to be low or moderate. The overall quality of evidence was based on the lowest quality of the primary outcome.

## Results

### Study Selection

**Figure 1:**
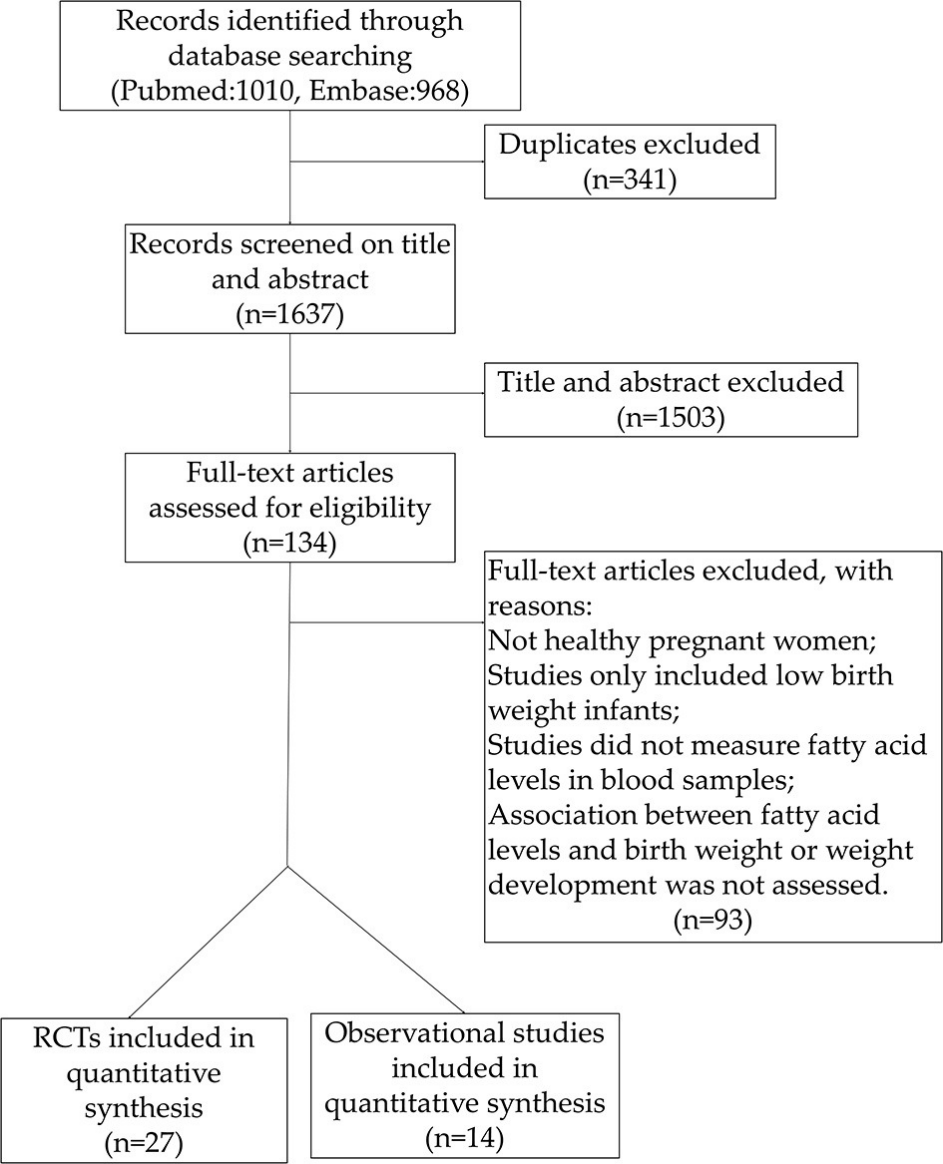
Study flow chart.

A total of 1978 studies were identified through the literature search, of which 341 articles were excluded due to duplication. A total of 1,596 articles were excluded during the title and abstract screening (*n* = 1,503) and full text assessments (*n* = 93), due to different reasons. Study selection results are shown in Figure 1.

### Randomized Controlled Trials

#### Description of Studies

A total of 27 studies representing 19 trials met the inclusion criteria, all of them investigating influences of DHA or DHA and EPA supplementation during healthy pregnancies on birth weight or weight in childhood (44–70). In our protocol, we included children up to 18 years of age, but no studies measured the effect of n-3 LCPUFA or TFAs on weight after 10 years of age. We also planned to explore relationships between body composition, fat distribution, gender differences, and sub-group analyses of risk of bias, but not enough eligible studies were included. The papers were based on a total of 19 RCTs, as eight of the papers undertook long-term follow-up of the children: DOMInO trial (51, 52), NUHEA (53, 54), INFAT (55, 56), COPSAC (57, 58), trials from Mexico (44, 45), trials from the USA (46, 47), trials from Germany (59, 60), and trials from the Netherlands (61, 62). A total of 6,408 infants were investigated in relation to birth weight; the numbers of children included in relation to weight and BMI at age 0–4 years were 2,097 and 1,908, respectively, and the numbers of children included in relation to weight and BMI at age 5–10 years were 3,477 and 3,445, respectively. The studies' characteristics are summarized in Table 1.

**Table 1:** Summary of DHA supplementation trials.

Authors, years (country)^*^ (primary paper)	Authors, years^*^ (companion papers)	**Intervention groups**	**Control groups**	**n-3 LCPUFA dose**	**Number of participants**		**Duration of intervention**	**Duration of anthropometric follow-up**
					**Intervention (boys %)**	**Control (boys %)**		
**DHA supplementation**								
Ramakrishnan et al. (44) (Mexico)	Gonzalez-Casanova et al. (45)	Algal DHA capsule	Olive oil capsule	400 mg/day	487 (46.1%)	486 (45.9%)	18–22 weeks of gestation until birth	At birth and 60 months
Carlson et al. (46) (USA)	Hidaka et al. (47)	Algal DHA capsule	A mix of soybean and corn oil capsule	600 mg/day	154 (49%)	147 (54%)	8–20 weeks of gestation until birth	At birth and 5 years
Mulder et al. (48) (Canada)		Algal DHA capsule	A mix of soybean and corn oil capsule	400 mg/day	104 (40.4%)	111 (55%)	Before 16 weeks of gestation until birth	At birth
Harris et al. (49) (USA)		Algal DHA capsule	Olive oil capsule	300 mg/day or 600 mg/day	107 117 (no information)	121 (no information)	16–20 weeks of gestation until birth	At birth
Smuts et al. (50) (USA)		DHA enriched eggs	Regular eggs	183.9 ± 71.4 mg/day (mean ± SD)	18 (no information)	16 (no information)	24–28 weeks of gestation until birth	At birth
**DHA+EPA supplementation**								
Makrides et al. (51) (Australia)	Muhlhausler et al. (52)	Fish oil capsule	Vegetable oil	800 mg/day DHA + 100 mg/day EPA	1,197 (50.1%)	1,202 (49.8%)	After 21 weeks of gestation until birth	At birth, and 3 and 5 years
Escolano-Margarit et al. (54) (Germany, Spain, Hungary)	Campoy et al. (53)	Fish oil-enriched milk	Milk	500 mg/day DHA + 150 mg/day EPA	43 (53.5%)	47 (61.7%)	20 weeks until birth	At birth, and 4 and 6.5 years
Hauner et al. (55) (Germany)	Brei et al. (56)	Fish oil capsule	Dietary counseling	1,020 mg/day DHA + 180 mg/day EPA	92 (52%)	96 (52%)	15 weeks of gestation to 16 weeks post-partum	At birth, 6 weeks, 4 months, 6 months, and 2, 3, 4, and 5 years
Vinding et al. (57) (Denmark)	Vinding et al. (58)	Fish oil capsule	Olive oil capsule	888 mg/day DHA + 1,320 mg/day EPA	304 (48.2%)	301 (53.1%)	24 weeks of gestation to 1 week post-partum	At birth and 6 years
Bergmann et al. (60) (Germany)	Bergmann et al. (59)	Combined supplement [fish oil + basic supplement + fructooligosaccharide (FOS)]	Combined supplement (basic supplement + FOS)	200 mg/day DHA + 60 mg/day EPA	41 (40%)	74 (47%)	Mid-pregnancy to 12 weeks post-partum	At birth, 21 months, and 6 years
Van Goor et al. (61) (Netherlands)	Van Goor et al. (62)	Fish oil capsule	Soybean oil capsule	220 mg/day DHA + 34 mg/day EPA	42 (39%)	36 (61.8%)	15.6–17.4 weeks of gestation to 12 weeks post-partum	At birth and 18 months
Judge et al. (63) (USA)		Fish oil-enriched cereal bar	Corn oil cereal bar	267 mg/day DHA + 34 mg/day EPA	27 (48.1%)	21 (66.7%)	24 weeks until birth	At birth
Keenan et al. (64) (USA)		Fish oil capsule	Soybean oil capsule	450 mg/day DHA + 90 mg/day EPA	34 (58.8%)	15 (46.7%)	6 weeks supply during pregnancy	At birth
Ostadrahimi et al. (65) (Iran)		Fish oil capsule	Liquid paraffin	120 mg/day DHA + 180 mg/day EPA	75 (56%)	75 (61.3%)	20 weeks of gestation to 4 weeks post-partum	At birth, 4 and 6 months
Hurtado et al. (66) (Spain)		Fish oil-enriched dairy	Dairy	320 mg/day DHA + 72 mg/day EPA	56 (52.6%)	54 (47.4%)	28 weeks until 16 weeks post-partum	At birth
Malcolm et al. (67) (UK)		Fish oil capsule	Sunflower oil capsule	200 mg/day DHA + 36 mg/day EPA	28 (52%)	27 (38%)	15 weeks of gestation until birth	At birth
Sanjurjo et al. (68) (Spain)		Dietary formula	Placebo (non-supplemented)	200 mg/day DHA + 40 mg/day EPA	8 (no information)	8 (no information)	26–27 weeks of gestation until birth	At birth
Helland et al. (69) (Norway)		Cod liver oil	Corn oil	1,183 mg/day DHA + 803 mg/day EPA	82 (43%)	61 (56%)	18 weeks of gestation to 12 weeks post-partum	At birth and 7 years
Olsen et al. (70) (Denmark)		Fish oil capsules	Olive oil capsules	920 mg/day DHA + 1,280 mg/day EPA	266 (no information)	136 (no information)	30 weeks of gestation until birth	At birth

* Primary paper (columns 1) and companion papers (columns 2) from one trial reported at different ages or outcomes; these publications were included in different meta-analyses.

*n-3 LCPUFA, n-3 long chain polyunsaturated fatty acid; DHA, Docosahexaenoic Acid; EPA, Eicosapentaenoic Acid*.

Among the 27 studies, most (*n* = 18 studies) were published after 2011 (45–49, 52–58, 60, 62–66), 8 studies were published the decade before (2000–2010) (44, 50, 51, 59, 61, 67–69), and 1 study was published in 1992 (70). One trial was from Australia (51, 52); one was from Asia (65); nine were from the USA, of which eight did not specify ethnicity (44–50, 63, 64); and one study only included African-American women (64). The remaining RCTs were from Europe (53–62, 66–70). The studies varied in size, ranging from 2,400 in the largest Australian RCT (51, 52) to only 16 participants in a Spanish RCT (54), but most of the studies included between 20 and 500 pregnant women.

Some of the studies limited inclusion by maternal pre-pregnancy weight in the range of 50–92 kg (53, 54) or pre-pregnancy BMI <30 kg/m^2^ (65), between 18 and 30 kg/m^2^ (55, 56, 66), or <40 kg/m^2^ (46, 47, 64). Nine studies excluded pregnant women with regular intake of marine supplements and other types of supplements (49, 51–56, 66, 70), or a high intake of fish (44, 45, 49, 64, 67, 70), milk (59, 60), or a history of drug use, smoking, or alcohol abuse (51, 52, 55, 56, 59, 60, 63, 64). Furthermore, two studies excluded pregnant women with vegetarian or vegan diets (61, 62).

Most of the studies used both DHA and EPA as supplements, with doses of supplementation ranging from around 200 to 2,200 mg/day n-3 LCPUFA. Seven studies used DHA as the single supplement with doses ranging from 200 to 600 mg per day (44–50). The majority of the trials used fish oil (44–49, 51, 52, 55–63, 65–67, 69, 70), but four trials supplied DHA or DHA and EPA with different foods such as milk, eggs, or cereal bars (50, 53, 54, 63, 66, 68). Most of the trials provided vegetable oil capsules to the participants in the control groups (44–49, 51, 52, 57, 58, 61–64, 67, 69, 70), and where relevant, they gave the controls standard food products, eggs (50), or milk (53, 54, 66). Only a few of the studies did not provide the control group with a comparator, but gave them only dietary counseling (55, 56).

Information on participants, supplementation, and duration of intervention and follow-up are described in Tables 1, 2.

**Table 2:** Summary of outcomes reported in the included RCTs.

**n-3 LCPUFA doses**	**Studies investigated birth**	**Studies investigated 0–4 years of age (age of measure)**	**Studies investigated 5–10 years of age (age of measure)**
**Weight**			
0–300 mg/day n-3 LCPUFA	Bergmann et al. (59), Van Goor et al. (61), Judge et al. (63), Ostadrahimi et al. (65), **Smuts et al**. **(****50****)**, Malcolm et al. (67), Sanjurjo et al. (68)	**(21 mohths) Bergmann et al**. **(****59****)**, (18 months) Van Goor et al. (62), (6 months) Ostadrahimi et al. (65)	(6 years) Bergmann et al. (60)
301–650 mg/day n-3 LCPUFA	Ramakrishnan et al. (44), Gonzalez-Casanova et al. (45), Escolano Margarit et al. (54), Campoy et al. (53), **Carlson et al**. **(****46****)**, Mulder et al. (48), Harris et al. (49), **Keenan et al**. **(****64****)**, Hurtado et al. (66)		(5 years) Gonzalez-Casanova et al. (45), (5 years) Hidaka et al. (47)
More than 650 mg/day n-3 LCPUFA	**Makrides et al**. **(****51****)**, **Hauner et al**. **(****55****)**, **Vinding et al**. **(****57****)**, Helland et al. (69), **Olsen et al**. **(****70****)**	(3 years) Muhlhausler et al. (52), (3 years) Brei et al. (56)	(5 years) Muhlhausler et al. (52), (5 years) Brei et al. (56), (6 years) Vinding et al. (58) (7 years) Helland et al. (69)
**BMI**			
0–300 mg/day n-3 LCPUFA	Bergmann et al. (59)	**(21 mohths) Bergmann et al**. **(****59****)**	(6 years) Bergmann et al. (60)
301–650 mg/day n-3 LCPUFA		(4 years) Escolano Margarit et al. (54)	(6.5 years) Campoy et al. (53)
More than 650 mg/day n-3 LCPUFA		(3 years) Muhlhausler et al. (52)	(5 years) Muhlhausler et al. (52), (7 years) Helland et al. (69)
**BMI z score**			
0–300 mg/day n-3 LCPUFA		(21 months) Bergmann et al. (60)	(6 years) Bergmann et al. (60)
301–650 mg/day n-3 LCPUFA			(5 years) Gonzalez-Casanova et al. (45), (5 years) Hidaka et al. (47)
More than 650 mg/day n-3 LCPUFA		(3 years) Muhlhausler et al. (52)	(5 years) Muhlhausler et al. (52), **(6 years) Vinding et al**. **(****58****)**
**Circumference (head, arm, Waits)**			
0–300 mg/day n-3 LCPUFA	Judge et al. (63), Ostadrahimi et al., (65), Smuts et al. (50), Malcolm et al. (67)	(18 months) Van Goor et al. (62), (6 months) Ostadrahimi et al. (65)	(6 years) Bergmann et al. (60),
301–650 mg/day n-3 LCPUFA	Ramakrishnan et al. (44), Gonzalez-Casanova et al. (45), **Carlson et al**. **(****46****)**, Harris et al. (49), Campoy et al. (53)		
More than 650 mg/day n-3 LCPUFA	Brei et al. (56), Vinding et al. (57)	(3 years) Muhlhausler et al. (52), (3 years) Brei et al. (56)	(5 years) Muhlhausler et al. (52), (5 years) Brei et al. (56), **(6 years) Vinding et al**. **(****58****)**
**BMI percentile**			
More than 650 mg/day n-3 LCPUFA		(3 years) Muhlhausler et al. (52), (3 years) Brei et al. (56)	(5 years) Muhlhausler et al. (52), (5 years) Brei et al. (56)
**Fat mass (g)/body fat (%)**			
More than 650 mg/day n-3 LCPUFA	Hauner et al. (55)	(3 years) Muhlhausler et al. (52), (3 years) Hauner et al. (55)	(5 years) Muhlhausler et al. (52), **(5 years) Hidaka et al**. **(****47**)^*^

* n-3 LCPUFA doses: 301–650 mg/day n-3 LCPUFA.

*Bold text: intervention groups showed higher result than control groups*.

#### Meta-Analysis Results From RCTs

**Figure 2:**
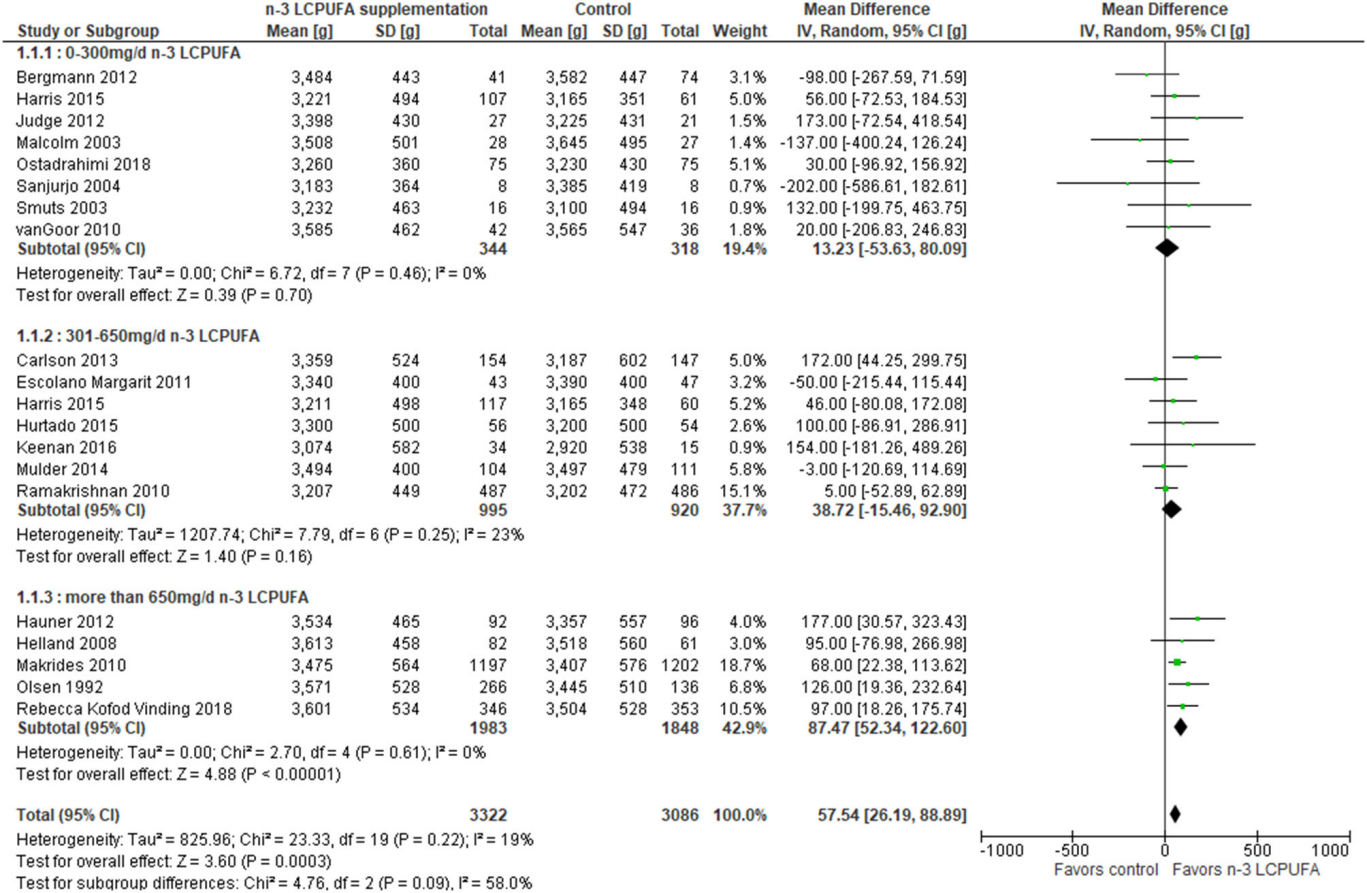
Maternal n-3 LCPUFA supplementation in pregnancy, dose of supplement given and offspring birth weight.

A total of 19 RCT studies were combined in a meta-analysis on birth weight (44, 46, 48–51, 54, 55, 57, 60, 61, 63–71). The meta-analysis showed significant overall higher birth weight in the n-3 LCPUFA-supplemented compared to the control groups, irrespective of intake level [mean differences (MD) 57.5 g, 95% CI 26.2–88.9, *n* = 6,408, *I*^2^ = 19%, Figure 2, Table 3]. Stratified analysis of the effects of n-3 LCPUFA intervention doses showed a higher birth weight among the supplemented individual with doses higher than 650 mg/day compared to the control groups, but not among individuals supplied with lower doses (MD 87.5 g, 95% CI 52.3–122.6, *n* = 3,831, Figure 2, Table 3).

**Table 3:** GRADE quality assessment.

**Outcomes**	Anticipated absolute effects^*^ (95% CI)	**Relative effect (95% CI)**	**No. of participants (studies)**	**Certainty of the evidence (GRADE)**	**Comments**
	**Risk with no n-3 LCPUFA** **Risk with n-3 LCPUFA**				
Birth weight (RCT)	The mean birth weight in the intervention was **57.5 g higher** (26.2–88.8 g)	MD **57.5 g higher** (26.2–88.8)	6,408 (19 RCTs)	⊕⊕⊕° MODERATE^a^	n-3 LCPUFA probably results in an increase in birth weight compared to controls groups
Weight at 0–4 years (RCT)	The mean weight at 0–4 years of age in the intervention group was **0.05 kg higher** (−0.27 to 0.18 kg)	MD **0.05 kg higher** (−0.27 to 0.18)	2,097 (6 RCTs)	⊕⊕°° LOW^a^^,^ ^c^	No evidence for an effect of n-3 LCPUFA on weight at 1–4 years compared to control
BMI at 0–4 years (RCT)	Standard mean difference at 0–4 years of age in the intervention group was **0.08 kg/m**^**2**^ **higher** (−0.28 to 0.44 kg/m^2^)	SMD **0.08 kg/m**^**2**^ **higher** (−0.28 to 0.44)	1,908 (4 RCTs)	⊕°°° VERY LOW^a^^,^ ^b^^,^ ^c^	No evidence for an effect of n-3 LCPUFA on BMI at 1–4 years compared to control
Weight at 5–10 years (RCT)	The mean weight at 5–10 years of age in the intervention group was **0.20 kg higher** (−0.05 to 0.45 kg)	MD **0.20 kg higher** (−0.05 to 0.45)	3,477 (7 RCTs)	⊕⊕⊕° MODERATE^c^	No evidence for an effect of n-3 LCPUFA on weight at 5–10 years compared to control
BMI at 5–10 years (RCT)	Standard mean difference at 5–10 years of age in the intervention group was **0.11 kg/m**^**2**^ **higher** (0.04–0.18 kg/m^2^)	SMD **0.11 kg/m**^**2**^ **higher** (0.04–0.18)	3,445 (7 RCTs)	⊕⊕⊕° MODERATE^a^	n-3 LCPUFA probably results in an increase in BMI compared to controls groups

* The risk in the intervention group (and its 95% CI) is based on the assumed risk in the comparison group and the relative effect of the intervention (and its 95% CI). CI, confidence interval; MD, mean difference; SMD, standardized mean difference. GRADE Working Group grades of evidence. High certainty: We are very confident that the true effect lies close to that of the estimate of the effect. Moderate certainty: We are moderately confident in the effect estimate: The true effect is likely to be close to the estimate of the effect, but there is a possibility that it is substantially different. Low certainty: Our confidence in the effect estimate is limited: The true effect may be substantially different from the estimate of the effect. Very low certainty: We have very little confidence in the effect estimate: The true effect is likely to be substantially different from the estimate of effect.

a High risk of bias for included studies.

b High heterogeneity.

c Wide confidence intervals.

*Bold values indicates our results*.

**Figure 3:**
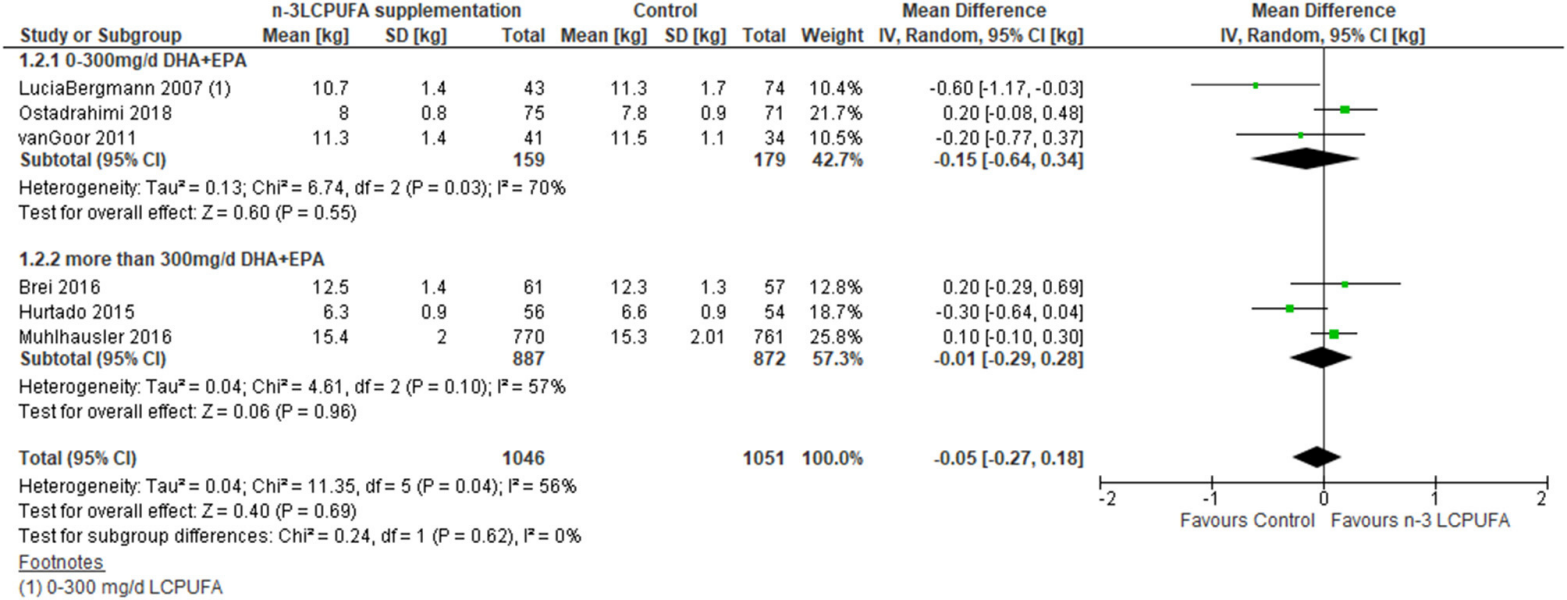
Maternal n-3 LCPUFA supplementation in pregnancy, dose given and offspring weight at age 0–4 years.

**Figure 4:**
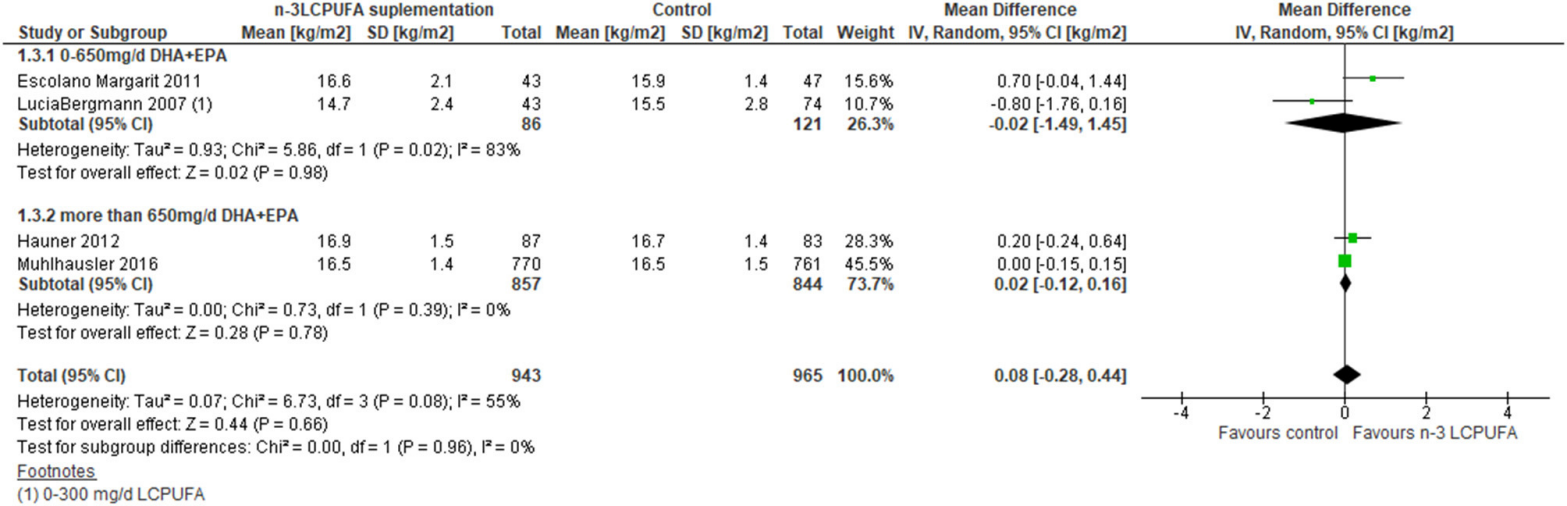
Maternal n-3 LCPUFA supplementation in pregnancy, dose given and offspring BMI at 0–4 years of age.

Eight RCT studies were included in the meta-analysis, which investigated supplementation of n-3 LCPUFA in pregnancy for development in weight or BMI at 0–4 years of age in the offspring (52, 54–56, 59, 62, 65, 66). The meta-analysis showed no difference in weight (MD −0.05 kg, 95% CI −0.3 to 0.2, *n* = 2,097, Figure 3, Table 3) or BMI (MD 0.08, 95% CI −0.3 to 0.4, *n* = 1,908, Figure 4, Table 3), irrespective of dose.

**Figure 5:**
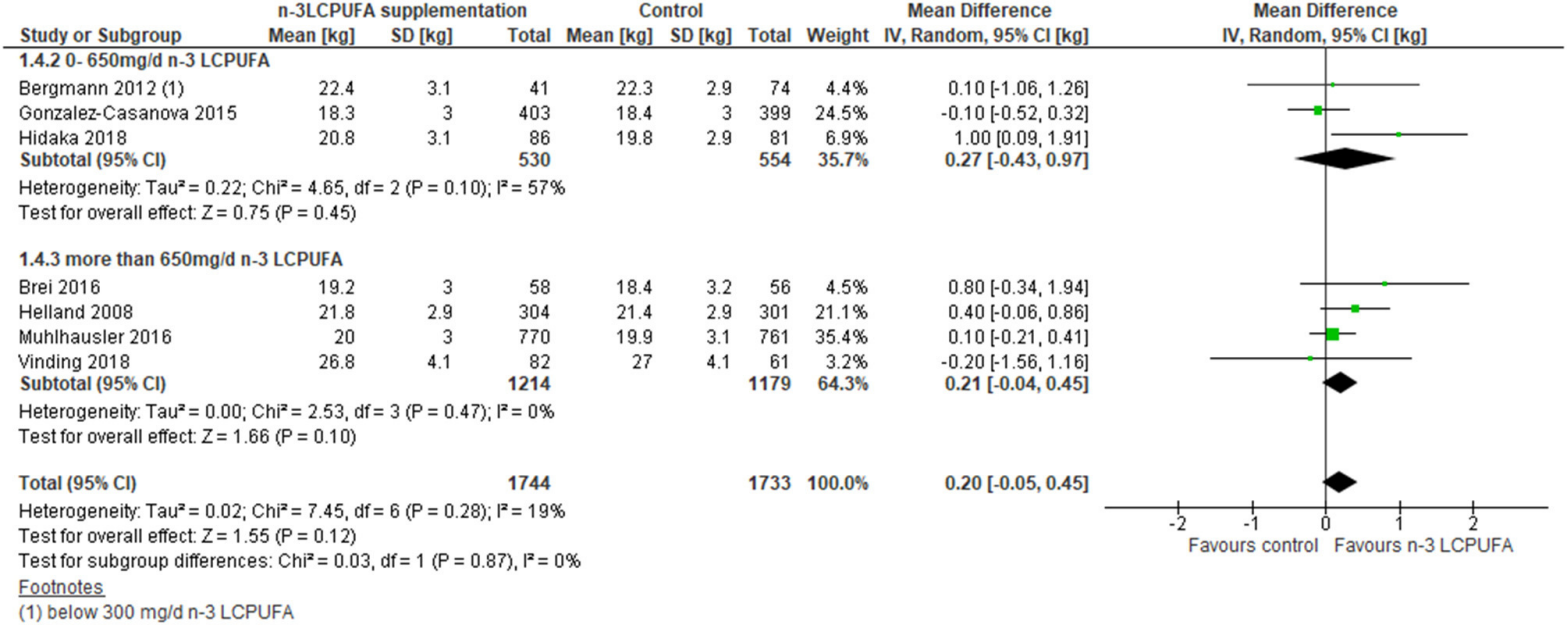
Maternal n-3 LCPUFA supplementation in pregnancy, dose and offspring weight at 5–10 years of age.

**Figure 6:**
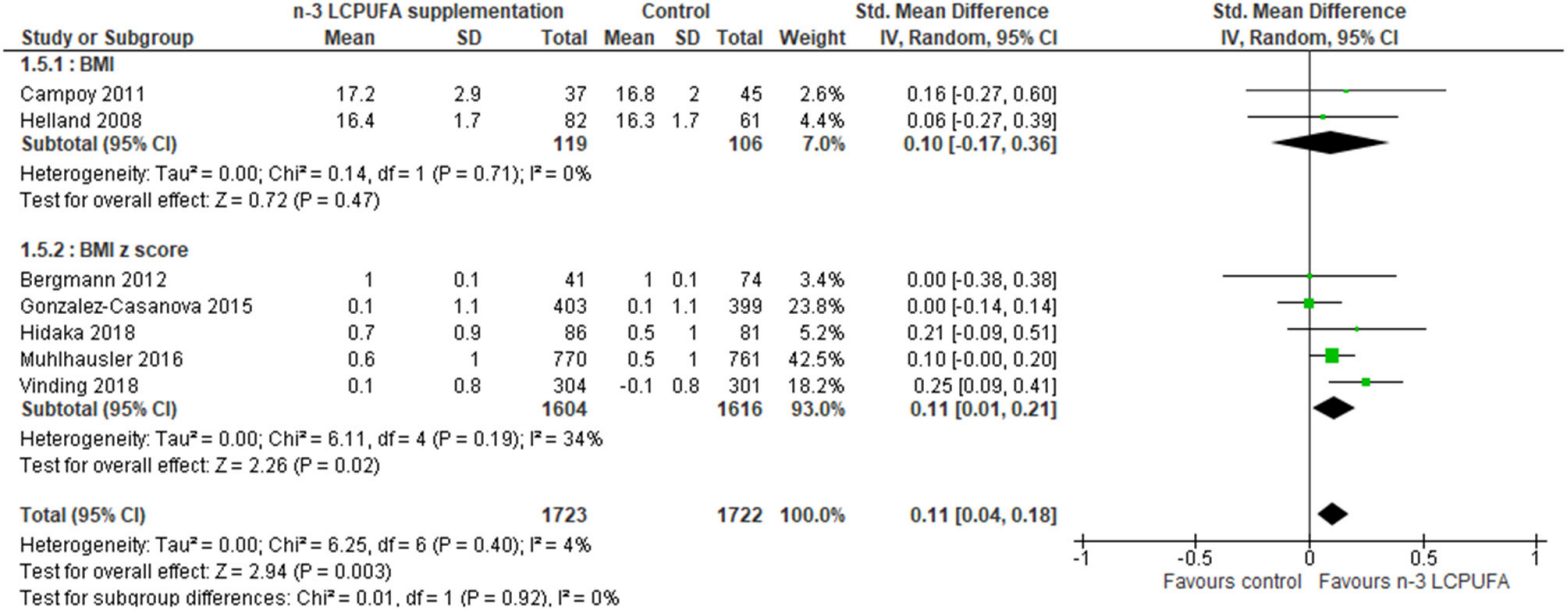
Maternal n-3 LCPUFA supplementation in pregnancy and offspring BMI or BMI Z score at 5–10 years of age.

Eight RCT studies investigated the importance of gestational DHA (45, 47) or DHA+EPA (52, 53, 56, 58, 60, 69) supplementation for offspring BMI, BMI *z* score, or weight at 5–10 years of age. No differences in weight development between the control and the n-3 LCPUFA-supplemented groups were found (Figure 5, Table 3). A difference in combined BMI and BMI *z* score was found where the n-3 LCPUFA intervention groups had slightly higher BMIs than the control groups (SMD 0.11, 95% CI 0.04–0.18, *n* = 3,445, Figure 6, Table 3).

A total of two trials (four papers) were not included in previous meta-analyses (57–60).

#### Grade

The overall quality of the meta-analysis results for birth weight and weight or BMI and BMI *z* scores at 5–10 years of age was rated as moderate quality, while the quality of meta-analysis results for weight at 0–4 years was rated as low and the results for BMI at 0–4 years was rated as very low quality (Table 3).

##### Risk of Bias

The risk of bias for all included RCTs was assessed by the Cochrane risk of bias tool (39) and is shown in Supplementary Table 1. The overall risk of bias was assessed; nine RCTs were rated as having low risk of bias (44, 45, 48, 51, 52, 57, 58, 64, 65), and nine RCTs were unclear in relation to allocation concealment, blinding of outcome assessment, or incomplete outcome data (46, 53, 54, 59–63, 68). Nine RCTs were found to have high risk of bias for more than one domain, and those articles were rated as having high risk of bias (47, 49, 50, 55, 56, 66, 67, 69, 70).

The most common issue in relation to risk of bias was treatment allocation concealment; 16 RCTs did not describe this issue (46, 47, 49, 50, 53, 56, 59–63, 66–69, 71). The method used for randomization sequence generation was unclear for six RCTs (45, 49, 63, 67, 70, 71) and another RCT did not use randomized methods (50). Four RCTs were rated as inadequate for blinding of patients and personnel or blinding of outcome assessors (50, 55, 56, 70).

The meta-analyses for birth weight, weight and BMI at 0–4 years of age were influenced by risk of bias. A total of 19 RCTs were included in the meta-analysis for birth weight. Of the 19 RCTs, 6 RCTs had low risk of bias (44, 48, 51, 57, 64, 65), 6 RCTs had unclear risk of bias (46, 54, 60, 61, 63, 68), and 7 RCTs had high risk of bias (49, 50, 55, 66, 67, 69, 70). In the five RCTs that investigated child weight at age 0–4 years, two RCTs had low risk of bias (52, 65), one had unclear risk of bias (59), and two had high risk of bias (56, 62). Four RCTs investigated child BMI at age 0–4 years, of which two RCTs had low risk of bias (52, 54), one had unclear risk of bias (59), and one had high risk of bias (55). The included RCTs were mainly affected by a lack of information about allocation concealment, a lack of blinding, or loss to follow-up.

##### Inconsistency

The quality of the meta-analysis for BMI at age 0–4 years was downgraded due to inconsistency (*I*^2^ = 53%). In this group, the countries, doses, and delivery methods varied (Figure 4). One study was conducted in Australia (52), and three were from Europe; two were from Germany (55, 59) and one study collected data from Germany, Spain, and Hungary (54). Moreover, the doses of n-3 LCPUFA differed, ranging from 260 mg/day (59) to 1,200 mg/day (55), using different delivery methods: fish oil plus fructo-oligosaccharides (59), milk (54), or fish oil (52, 55). Heterogeneity for all analysis groups was not affected by stratified analysis (Table 3).

##### Imprecision

Meta-analyses for weight or BMI at age 0–4 years and weight at age 5–10 years were downgraded due to wide confidence intervals (Table 3).

##### Publication Bias

It was only possible to assess publication bias for the meta-analysis for birth weight. Here, the funnel plot showed no indication of publication bias (Supplementary Figure 2, Table 3).

### Observational Studies

#### Description of Studies and Participants

In our protocol, we included children up to 18 years of age, but only one observational study investigated the effect of PUFAs on BMI up to 23 years of age (72). In total, 14 eligible observational studies investigated the relationship between blood levels of TFAs or n-3 LCPUFA during pregnancy or from cord blood or placental tissues, in relation to birth weight or weight in childhood (32, 33, 72–82). All the included observational studies are summarized in Table 4. A total of 11 of the studies were published after 2012 (72–78, 80–83), whereas three of the studies were published more than a decade ago (32, 33, 79). Overall, mean maternal age ranged from 29 to 33 years. Only one study included more multiparous women than nulliparous (83). Most of the studies included slightly more boys than girls ranging from 50 to 54% (32, 72–82), and one study included slightly more girls than boys (48.8%) (33).

**Table 4:** Summary characteristics of included observational studies (only DHA was included in the meta-analysis).

**Author, year, (country)**	**Sample tissue, sample time and participant (*n*, boys %)**	**Type of fatty acid**	**Confounders adjusted for**	**Results**
Meher et al. (73) (India)	Placental tissues (at delivery) (*n* = 64, no information)	DHA	BMI, age, and gestation age	Low placenta DHA in low-birth-weight infants.
Vidakovic et al. (74) (Netherlands) Jelena Vidakovic et al. (75) Grootendorst et al. (76) Grootendorst et al. (77)	Maternal plasma glyceroPL in week 20.5 (median) (*n* = 4,830, 50%)	DHA, EPA, TFA	BMI, smoking, gestational age at maternal blood sampling, child gender, maternal age, parity, educational level, alcohol use, folic acid supplement use, psychological symptoms and pregnancy complication, region.	High n-3:n-6 PUFA ratio associated with birth weight (β = 0.76 SD score, 95% CI 0.22, 1.29, *P* = 0.006). Maternal DHA and EPA levels not associated with BMI at 24 months or 6 years of age. High t18:1 was associated with low BW (β: −0.10; 95% CI: −0.15, −0.04; *P* < 0.001).
Bernard et al. (78) (Singapore)	Maternal plasma glyceroPL in weeks 26–28 (*n* = 979, no information)	DHA	Study center, ethnicity, child's sex, parity, fasting glucose levels, vitamin D levels, gestational weight gain at 26–28 weeks' gestation, maternal height and pre-pregnancy BMI, paternal height, familial income, maternal education and age.	High DHA not associated with child weight and BMI from birth to 5 years of age.
Elias et al. (32) (Canada)	Maternal and cord plasma TAG, PL, and CE in week 35 (*n* = 70, no information)	TFAs, EPA, DHA	No information	Maternal TFAs and n-3 LCPUFA were not associated with BW. TFAs were not related to the length of birth weight.
van Eijsden et al. (33) (the Netherlands)	Maternal plasma PL in week 13.5 ± 3.3 (*n* = 3,704, 48.8%)	EPA, DPA, DHA, TFAs	No	High TFA and low n-3 LCPUFA were associated with low BW (−52 to −172 g and −56 to −172 g, respectively).
Dirix et al. (79) (the Netherlands)	Umbilical vein plasma PL (and red blood cell) at birth (*n* = 730, 53.7%)	DHA, TFAs	BMI at study entry, maternal height, parity, alcohol during pregnancy, weight gain during pregnancy, gestational age, infant sex.	High plasma DHA and RBC PL TFA were associated with low BW (*P* < 0.001, *R*^2^ = 0.424, *r*^2^ = 0.033 and *P* = 0.004, *R*^2^ = 0.334, *r*^2^ = 0.058, respectively).
Moon et al. (80) (UK)	Maternal plasma in week 34 (*n* = 293, 52.2%)	n-3 and n-6 PUFA	Social class and highest educational qualification, pre-pregnancy body mass index, smoking status in late pregnancy, maternal age at delivery, parity, gestational weight, walking speed in late pregnancy, maternal mean daily intake of protein, fat and carbohydrate at 34 weeks gestation, the child's height and duration of breastfeeding.	n-3 LCPUFA correlated with high offspring lean mass at 4 and 6 years of age, but this was confounded by offspring height.
Stratakis et al. (72) (the Netherlands)	Umbilical vein plasma PL at birth (*n* = 250, 54.8%)	ALA, EPA+DHA n-3 LCPUFA	Maternal BMI at study entry, maternal smoking in pregnancy, parental education, child age terms (age^0.5^, age, age^3^), maternal age at birth, gestational weight gain, maternal alcohol intake in pregnancy, parity, breastfeeding status, and cross-products of each PUFA exposure with the child age terms.	No association between n-3 LCPUFA and BMI at 6 months to 23 years in both sexes. High n-3 LCPUFA was associated with length in male infants (β = 0.44, 95% CI = 0.07–0.82). Higher n-3:n-6 PUFA ratio was associated with high infant length in both sexes (β = 0.40, 95% CI = 0.01–0.78 and 0.42, 95% CI = 0.05–0.79 for males and females, respectively).
Cinelli et al. (81) (Italy)	Cord blood and maternal blood erythrocyte membranes at 12–24 h before birth (*n* = 607, 52.1%)	n-3 LCPUFA, DHA	Gestational weight gain, pre-pregnancy BMI, smoking, maternal age, offspring sex, gestational age, and parity.	High maternal and fetal DHA related to high birth weight (coefficient = 29.73, *P* = 0.007 and coefficient = −22.82, *P* = 0.037) and high maternal and fetal EPA related to long gestation (*P* = 0.001 and *P* = 0.039).
Kitamura et al. (82) (Japan)	Maternal venous blood at 36–38 weeks and cord venous blood (erythrocyte membranes) (*n* = 212, no information)	EPA, DHA, DPA, n-3LCPUFA	No	Maternal n-3 LCPUFA related to gestation length (EPA: *r* = 0.162, *P* = 0.018, DHA: *r* = 0.188, *P* = 0.006). n-3 LCPUFA was not associated with birth weight.
Maslova et al. (83) (USA)	Maternal and cord erythrocyte and plasma (*n* = 1,418, 51.3%)	EPA, DHA, EPA+DHA intake	Household income; pre-pregnancy BMI, smoking in pregnancy, maternal age; parity; maternal education (≥ and < college graduate); and offspring age and sex. Race/ethnicity was not included as we have previously shown that maternal fatty acid status was similar across race/ethnicity strata; 34 in a sensitivity analysis, adding race/ethnicity to the models did not change the results. Intermediary covariates (gestational weight gain, birth weight, and gestational age) were excluded to avoid over adjustment.	High maternal DHA+EPA and fish intake associated with low adiponectin at 3.2 years. High cord plasma DHA associated with low BMI z score and leptin at 3.2 years of age.

*BW, birth weight; PL, phospholipid*.

Information on participants and time of fatty acid measurements are given in Tables 4, 5.

**Table 5:** Summary of outcomes and fatty acids for all 14 included observational studies.

**Outcome**	**Studies only measured DHA**	**Studies measured DHA and EPA**	**Studies measured DHA and other PUFA**
Birth weight	Meher et al. (73) Bernard et al. (78) Dirix et al. (79)^*^	Elias et al. (32)^*^	Grootendorst et al. (76) van Eijsden et al. (33)^*^ Cinelli et al. (81) Kitamura et al. (82)
Weight at 1–4 years of age	Bernard et al. (78)		Moon et al. (80)
BMI at 1–4 years of age	Bernard et al. (78)	Maslova et al. (83)	Jelena Vidakovic et al. (75) Stratakis et al. (72)
Weight at 5–10 years of age	Bernard et al. (78)		Moon et al. (80)
BMI at 5–10 years of age	Bernard et al. (78)	Maslova et al. (83)	Vidakovic et al. (74) Stratakis et al. (72)

* *This study also measured TFA; another study only measured TFA (77)*.

It was not possible to conduct a meta-analysis due to the fact that included studies used different statistical methods and different units for fatty acid levels in blood or tissue samples. All the observational studies included in this systematic review investigated different kinds of fatty acids (DHA, EPA, and TFAs) and outcomes (weight and BMI). Four of the studies reported fatty acid levels in mg/L or μg/ml (33, 77, 80, 83), other studies presented in relative percentage (%, w/w) to total fatty acid levels (32, 72, 73, 76, 78, 79, 81, 82), one reported fatty acid levels in both ways (74, 75). Overall, four studies were from the Generation R Study from the Netherlands (74–77), three other studies also involved participants from the Netherlands (33, 72, 79), one study was from the UK (80), one study was from Italy (81), three studies were from Asia (73, 78, 82), one was from Canada (32), and, lastly, one study was from the USA [(83); Table 4].

#### Results From the Observational Studies

##### Birth Weight

In total, eight studies examined the relationship between different fatty acids and birth weight [(32, 33, 73, 76, 78, 79, 81, 82); Table 4]. Three studies showed contradicting results as two studies found that a low maternal n-3 LCPUFA level was related to lower birth weight (33, 81), whereas another study showed that a high fetal DHA level was associated with low birth weight (81). One other study found that high n-3 LCPUFA status was related to lower birth weight (79), and one study found no association between n-3 LCPUFA and birth weight (82). DHA levels ranged from 3.34 to 5.76% and EPA levels ranged from 0.50 to 0.77%.

Four studies investigated TFAs and birth weight (32, 33, 77, 79). Three studies found that high levels of TFAs during pregnancy were related to a lower weight at birth (33, 77, 79), while one study found no association between TFAs and birth weight (32) Two studies calculated regression coefficients and showed the relation between maternal levels of different fatty acids and birth weight (76, 78).

##### BMI at 0–4 Years of Age

Five observational studies investigated the association between n-3 LCPUFA from maternal or neonatal blood and weight or BMI at 0–4 years of age (72, 75, 78, 80, 83). Four of them used regression coefficients, two measured fatty acids in μg/ml, and the DHA level ranged from 51.8 to 88.9 μg/ml and mean EPA levels were 5.1–10.9 μg/ml (80, 83), while the other two studies measured fatty acids in weight percentage (wt%), and DHA levels ranged from 4.69 to 5.0 wt% and mean EPA levels ranged from 0.6 to 0.69 wt% (74, 78). The Moon et al. study investigated both DHA and EPA in relation to weight at 4 and 6 years of age and showed no association between maternal DHA and EPA level and BMI (80). Maslova et al. investigated both DHA and EPA in relation to BMI at age 0–4 years and showed that high DHA was associated with lower BMI *z* score (83).

No studies investigated the relation between TFA and weight or BMI at 0–4 years of age.

##### BMI at 5–10 Years of Age

A total of five studies investigated the relationship between n-3 LCPUFA from maternal and neonatal blood and weight or BMI at 5–10 years of age (72, 74, 78, 80, 83). Three studies showed no relationship between maternal n-3 LCPUFA status and weight (80) or BMI (72, 83) during childhood. One of these studies followed children from 6 months until 23 years of age, and the results showed no association between n-3 LCPUFA and BMI from infancy until young adulthood in boys and girls (72). Four studies used regression coefficients, and two of them measured fatty acids in μg/ml (80, 83), while the other two measured fatty acids in wt% (74, 78).

No studies investigated the relation between TFA and weight or BMI at 5–10 years of age.

## Discussion

The present meta-analysis of the RCTs showed that offspring birth weight was higher for n-3 LCPUFA-supplemented than for placebo-supplemented pregnant women, especially when supplementation dose was high (>650 mg/day). In addition, the supplementation with n-3 LCPUFA during pregnancy resulted in a modest increase in the BMI *z* score but had no effect on BMI of the offspring at 5–10 years of age. BMI varies with age and sex in children, while these variations are accounted for when BMI *z* scores are being used, which may explain why associations were seen only for BMI *z* scores (84). Furthermore, this result was rated moderate quality only, according to GRADE guidelines.

Eight previous systematic reviews have investigated the effect of n-3 LCPUFA supplementation during pregnancy/lactation for child growth (12, 20–26). In our meta-analysis, we further included two RCTs (four papers) that had not been included in any of the previous meta-analyses (57–60). Furthermore, we restricted our search to include RCTs giving supplements to healthy pregnant women during pregnancy only. We found n-3 LCPUFA supplementation to result in a higher birth weight, a finding that is in agreement with results from four of the eight previously published systematic reviews (12, 20, 21, 26). Our finding of an effect of maternal n-3 LCPUFA supplementation on BMI children aged 5–10 years is, however, not supported by any of the previous reviews. Indeed, three previous reviews found no support for an association when they investigated the effects of n-3 LCPUFA supplementation during pregnancy and/or lactation for childhood BMI (12, 24, 25). The conflicting results do not seem to depend on the period of when supplementation was provided, but the reviews differed somewhat with respect to other selection criteria, especially to whether or not high risk or only healthy pregnant women were included.

We made some additional analyses to explore potential effect modifiers including the importance of the dose of n-3 LCPUFA, which provided important information to pursue in future RCTs. The relationship between n-3 LCPUFA and both birth weight and later BMI appear to be dose-dependent, and no effects could be detected with doses <650 mg/day. This is not surprising as a dose–response relationship is expected, but it indicates that the n-3 LCPUFA intake has to be high in order to give rise to a relevant effect size. A dose >650 mg/day of n-3 LCPUFA is high compared to the intake supplied by the diet in most countries, but it is nevertheless feasible to achieve with a fish intake in accordance with the recommendations in many countries. Pregnant women are typically recommended to have a daily intake of 300 mg of n-3 LCPUFA, which, according to this review, might not be optimal in relation to the birth weight of the baby. However, the results from our analysis showed a mean difference in birth weight of 57.5 g between the n-3 LCPUFA-supplemented groups and the control groups and a mean difference in BMI at 5–10 years of 0.09 kg/m^2^, both of which may not be of major clinical relevance. In this regard, it has been reported that a higher birth weight of 586 g was related to a 25% higher odds of overweight at 15–20 years of age (OR 1.25, 95% CI 1.06–1.48) and that a 1.83 kg/m^2^ higher BMI at 5–7 years of age was also related to a high odds of overweight (OR 3.23, 95% CI 2.56–4.07) (85).

### Summary of Results From Observational Studies in Relation to Other Studies in the Field

Observational studies showed contradicting results relating maternal n-3 LCPUFA to low birth. Indeed, one found that low maternal n-3 LCPUFA was related to low birth weight and another found an association between high n-3 LCPUFA status at birth and low birth weight (79, 81). One study found no association (82). Three studies showed no relationship between maternal n-3 LCPUFA and weight (80) or BMI (72, 83) during childhood, and in addition, one of these studies did not find an association with BMI measured at different times between ages 6 months and 23 years (72).

For the four observational studies that investigated maternal TFA intake and birth weight (32, 33, 77, 79), the results from three studies from the Netherlands indicated that a high maternal TFA level was related to <50 g lower birth weight (33, 77, 79), while one study from Canada showed no association between maternal TFA levels and birth weight (32). TFA are high in stick margarine, cakes, and French fries and occur naturally in meat and some milk products (86, 87). As many countries have legislations on the allowed TFA level, decreases in TFA content in the diet have been observed over the past one to two decades (88, 89). A previous study have reported that the individual effects on cardiometabolic risk and lipid profile of different sources of TFA are contentious (27). However, the studies included in this review did not report the sources of TFA. TFAs have been shown to cross the placenta (90) and may influence both PUFA transfer and metabolism (34, 91). One study, which estimated maternal consumption of TFAs in the first two trimesters by food frequency questionnaire (34), found that a high maternal TFA intake, particularly 16:1t during weeks 13–28 of pregnancy, was associated with a high fetal growth *z* score. The authors argued that biomarkers may possibly reflect short-term intake while the assessed dietary intake of TFAs may be a better measure of long-term exposure (34).

### Comparing Results From RCTs and Observational Studies

The RCTs showed that n-3 LCPUFA was related to a higher birth weight and to a higher BMI at 5–10 years, while the observational studies generally showed no relationship between n-3 LCPUFA and birth weight or BMI. The discrepant results may be related to selection bias and confounding (92), which can influence the relationship between n-3 LCPUFA levels and growth and may have been present in the observational studies but generally not in the RCT. Thus, since RCTs are able to control for selection and measure biases, RCTs generally offer stronger evidential support than observational studies alone (93). However, even if sample sizing in RCTs are determined by balancing feasibility and power of the study (94). RCTs have also been criticized for including small and selected sub-groups (95), which may hamper generalizability (92). Therefore, large and diverse observational cohort studies have been encouraged (96).

One further aspect that needs to be considered in the comparison of the results from the meta-analysis of the RCTs and the observational studies is dose. In our meta-analysis of RCTs, no effects were shown with doses of <650 mg/day n-3 LCPUFA supplementation. One review showed that the average n-3 LCPUFA intake in observational studies has been reported to be 75–149 mg/day in the USA and Canada, 250–550 mg/day in Europe, and >550 mg/day in Japan and Singapore (97), suggesting that intake doses may be too low to increase birth weight.

Finally, it should be noted that the RCTs included in the present review were of moderate GRADE quality only, because of risk of bias, and the results of the present analysis may further have been affected by publication bias and heterogeneity. Hence, the present meta-results should be interpreted with some caution.

### n-3 LCPUFA Intake and Status in Observational Studies

The different results of the studies may also depend on the baseline levels of n-3 LCPUFA; e.g., if this level is high, there may be less room for improvement, and it may therefore be more difficult to find a significant difference even if high-dose supplements were given. Indeed, this review included studies from various countries, and it is well-known that intake and status of n-3 LCPUFA varies greatly between countries (98). The highest seafood n-3 fatty acid intake was found in Pacific Island nations, Denmark, South Korea, and Japan (97). These nations also had the highest levels of DHA and EPA measured from blood samples (99). Populations from Germany, Spain, the Netherlands, Singapore, and Australia were found to have low EPA+DHA blood levels, while levels were very low in Canada, the USA, Ireland, the UK, Italy, and Iran (99). Therefore, even if studies supplemented the same doses, they may still compare intervention and control groups, which are very different in terms of n-3 LCPUFA status.

The assessment of the importance of dose is further affected by the tissue sample used for evaluation of intake and status, e.g., fatty acid levels from different bio-materials seem to vary, and the level of DHA in cord plasma is higher than that in maternal plasma (100), while EPA status in infant plasma seems lower than that in maternal plasma (101). In the 14 included studies, three different tissues were used for extraction of lipids for the biomarker analysis. The most widely used tissue sample was plasma (32, 33, 72, 74–80), where lipid concentrations reflect recent maternal fat intake (24), as is probably also the case in the one study that used blood from the placenta (73). Three studies used erythrocytes as a bio-material (81–83), and this is considered to reflect long-term intake (24).

Blood samples in the studies included were collected at different times, ranging from 20.5 weeks into pregnancy to birth, and since it is well-known that both maternal erythrocytes and plasma DHA composition may decrease from the second trimester to delivery (102), studies may not be comparable. Also, the relative levels (% wt/wt) of n-3 LCPUFA decline throughout pregnancy (103). All of the five observational studies included in the meta-analysis presented fatty acids in relative levels; four of them extracted fatty acids from plasma around mid-pregnancy, and one used the placenta at birth, hampering comparison.

### Maternal n-3 LCPUFA Supplementation

DHA is transferred through the placenta and is rapidly stored in the fetal brain and retinal tissue (104). The fetus's need for nutrients is greater during the last trimester of pregnancy due to a rapid development of the brain and retina in the third trimester, and n-3 LCPUFA may therefore be most essential between the third trimester and 18 months of life (13). It has been suggested that the requirement in the third trimester of pregnancy is approximately 50 mg/(kg/day) n-3 PUFA and 400 mg/(kg/day) n-6 PUFA (105). It is therefore assumed that the period of supplementation or the assessment of maternal intake and status is relevant to consider in the evaluation of the results. All included RCTs started before the third trimester. The majority of the RCTs started supplementation around 20 weeks of gestation (44, 45, 49, 53, 54, 59, 60, 65, 69) and five studies started earlier, from 8 weeks or before 17 weeks (48, 55, 61, 62, 67). Only two RCTs started at 28 or 30 weeks of gestation. One RCT did not give information about when they started the intervention, but informed that they supplied n-3 LCPUFA for 6 weeks during pregnancy (64). In general, supplementation stopped at delivery, but 11 studies continued post-partum (55–62, 65, 66, 69). Jointly, the results indicate that dose as well as period of n-3 LCPUFA supplementation is important to consider.

It is also relevant to consider the comparator in RCTs. The different RCTs that were included in this review provided different sources of n-3 LCPUFA as supplements to participants. Different sources of oils have different quantity and quality of n-3 PUFAs or a different DHA:EPA ratio (106, 107). A previous study suggested that DHA from an algal oil supplement can replace a fish oil supplement (108), but another study found n-3 PUFA from plant oil is low in EPA and DHA (109). Some studies used PUFA enriched foods as supplements, which may significantly increase the fat profile and level in the human body (110). However, these kinds of foods required special storage conditions, since n-3 LCPUFA may oxidize to toxic peroxides (110). The fatty acid composition can also result in difference in the contribution to infant growth and body composition (111). The supplemented fish oils or seed oils contain some saturated fatty acids (up to 30–40%) and even TFA (112, 113), which may explain some of the difference in the contribution to the metabolism in the pregnant woman and potentially contribute to associations with birth weight or childhood growth.

### Advantages and Limitations

It is a strength of the present review that it is based on a large number of studies, that the included studies (both RCTs and observational studies) were identified from four continents and included 15 different countries. Since it has been suggested that BMI is not an accurate marker for obesity in children (114), the present meta-analysis included the results for weight, BMI, as well as BMI *z* score. We collected data at different childhood ages, from birth to 10 years of age, enabling us to investigate the effects of n-3 LCPUFA and TFA throughout childhood.

On the other hand, most of the RCTs were from Europe, and data from some countries were still missed. Only one RCT was from Asia (65), and two RCTs were from Australia (51, 52), limiting generalization to other populations than mainly Caucasians. In addition, only two RCTs were conducted in a Newly Industrialized Country [Mexico (44, 45)] or a developing country [Iran (65)]. Various tissues were used to measure fatty acid levels, which will reflect maternal fatty acid intake to different extents.

## Conclusion

In conclusion, this review and meta-analysis does support a relationship between maternal or neonatal n-3 fatty acid levels and offspring birth weight and weight development into childhood. This was specifically the case for high doses of DHA and/or EPA supplementation of >650 mg/day, whereas no association was found between low doses of supplementation and weight development. Furthermore, we found some suggestion that high TFA levels during pregnancy may be related to a risk of low birth weight, while studies examining relations between TFA levels during pregnancy and infant or child weight were generally absent. Also, all effect sizes were relatively small and may not be considered clinically relevant. In the present review, we have analyzed results not only from RCTs but also from observational studies. Results from RCTs were rated as moderate quality. Thus, more high-quality long-term studies are still needed, especially studies that investigate high-dose supplementation during pregnancy of n-3 LCPUFA, effects of TFA, long-term follow-up in relation to infant and childhood weight and growth, as well as more studies from Asia.

## Data Availability Statement

The original contributions presented in the study are included in the article/Supplementary Material, further inquiries can be directed to the corresponding author/s.

## Author Contributions

XR contributed to the original draft. BV, JR, KW, SR, LL, BH, and IS contributed to methodology and critically reviewed this paper. All authors have read and agreed to the published version of the manuscript. All the authors contributed to the development of this paper.

## Conflict of Interest

The authors declare that the research was conducted in the absence of any commercial or financial relationships that could be construed as a potential conflict of interest.
